# Chromosome-Level Genome Assembly of Trichoderma cornu-damae Using Hi-C Data

**DOI:** 10.1128/mra.00142-22

**Published:** 2022-10-05

**Authors:** Sung-Eun Cho, Young-Nam Kwag, Dong-Hyeon Lee, Chang Sun Kim

**Affiliations:** a Forest Biodiversity Division, Korea National Arboretum, Pocheon, South Korea; b Division of Forest Insect Pests and Diseases, National Institute of Forest Science, Seoul, South Korea; University of California, Riverside

## Abstract

Trichoderma cornu-damae is known as a poisonous mushroom (Basidiomycota, Fungi) that produces several trichothecene mycotoxins. We constructed a chromosome-level genome assembly of T. cornu-damae with Hi-C sequencing data.

## ANNOUNCEMENT

Trichoderma cornu-damae (Basidiomycota, Fungi) is known to grow in Republic of Korea, Japan, China, and Indonesia (1, 2). It is shaped like a deer’s red horn, but in its immature period it resembles Ganoderma lucidum or Cordyceps militaris, which are well known as edible or medicinal mushrooms. Trichothecenes are powerful protein synthesis inhibitors, and tissues containing cells that are actively and rapidly growing and dividing are susceptible to these toxins (2).

**FIG 1:**
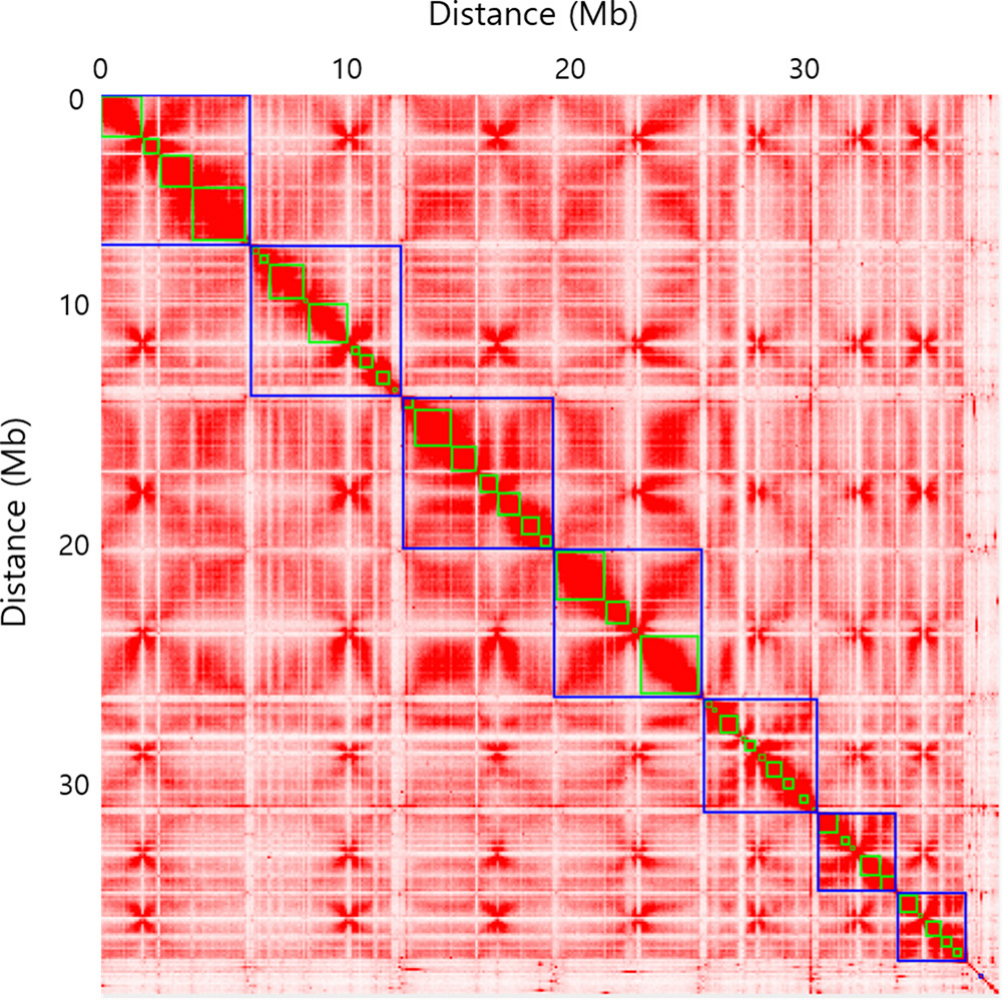
Hi-C interaction heatmap for each of the 7 chromosomes of Trichoderma cornu-damae. Distinct chromosomes are highlighted by blue boxes, and homologous chromosome pairs are numbered.

A genomic DNA library for next-generation sequencing (NGS) was constructed according to a standard Illumina paired-end (PE) library protocol and sequenced (150-bp PE reads) using the Illumina HiSeq X platform. An Oxford Nanopore Technologies (ONT) Nanopore sequencing library was constructed using the 1D ligation sequencing kit (SQK-LSK109; ONT). Raw Nanopore sequencing data (4.2 Gb) were trimmed to 3.8 Gb. The trimmed Nanopore data were *de novo* assembled using NextDenovo2.3.0 with a minimum read length of 1,000 bp and a seed length of 10,107 bp. Hi-C data were generated using a Proximo Hi-C 2.0 kit (Phase Genomics, Seattle, WA) (3). Briefly, T. cornu-damae cells were cross-linked using a formaldehyde solution, digested using the DpnII restriction enzyme, and proximity ligated with biotinylated nucleotides. A total of 169,680,565 read pairs were generated and aligned to the draft assembly using BWA-MEM (4) with the −5SP and –t 8 options. SAMBLASTER (5) was used to flag PCR duplicates, and nonprimary and secondary alignments were removed with SAMtools (6) using –F 2304 filtering. A total of 36 breaks in 25 contigs were introduced, and the same alignment procedure was repeated from the beginning with the resulting corrected assembly. Chromosome-scale scaffolds were generated by the Proximo Hi-C genome scaffolding platform (Phase Genomics) (7). Approximately 40,000 separate Proximo runs were performed to optimize the number of scaffolds and scaffold construction in order to make the scaffolds concordant with the observed Hi-C data (8). This process resulted in a set of 7 chromosome-scale scaffolds containing 37.3 Mbp of sequence (96.6% of the input assembly) (Fig. 1). Finally, Juicebox (9, 10) was used to correct scaffolding errors and to introduce 36 breaks into 25 putative misjoined contigs, resulting in a final set of 7 scaffolds spanning 37.3 Mbp, with a scaffold *N*_50_ value of 6.5 Mbp. The GC content of the genome is 43.15%.

Repeat families of T. cornu-damae were established by RepeatModeler v1.0.11, and then repetitive sequences were masked with RepeatMasker v4.0.5. Briefly, first gene prediction was conducted with transcripts of seven reported *Trichoderma* species and a protein set of 17 *Hypocreales* species, and then an *ab initio* training data set was constructed by GeneMark-ES v4.38 (11), SNAP v2006-07-28 (12), and AUGUSTUS v3.3.4 (13) with a first gene prediction result with an annotation edit distance (AED) cutoff value of <0.25. Final genes were predicted via the first gene prediction result and the *ab initio* training data set with Maker and EVidenceModeler v1.1.1 (14), which resulted in 8,544 genes. A Benchmarking Universal Single-Copy Orthologs (BUSCO) v4.0.2 analysis (15) was used for evaluation of the predicted gene set with the fungi_odb10 data set. Complete BUSCOs were calculated as 97.6%, with 95.9% single-copy BUSCOS and 1.7% duplicated BUSCOs.

### Data availability.

This whole-genome shotgun project has been deposited in DDBJ/ENA/GenBank under accession number JAIWOZ000000000. The version described in this paper is version JAIWOZ010000000. The Nanopore sequencing data are available under SRA accession number SRR18250172. The Illumina (NGS sequencing) and Hi-C data are available under SRA accession numbers SRR18250171 and SRR18250170, respectively. The BioProject accession number is PRJNA739541, and the BioSample accession number is SAMN19791815.
